# Effects of somatosensory stimulation on corticomotor excitability in patients with unilateral cerebellar infarcts and healthy subjects - preliminary results

**DOI:** 10.1186/s40673-014-0016-5

**Published:** 2014-11-05

**Authors:** Suzete Nascimento Farias da Guarda, Adriana Bastos Conforto

**Affiliations:** Hospital das Clínicas/São Paulo University, São Paulo, Brazil; Hospital São Rafael, Salvador, Brazil; Instituto Israelita de Ensino e Pesquisa Albert Einstein, São Paulo, Brazil; Rua Waldemar Falcão n 1547 ap 1201 Horto Florestal 40.295-010, Salvador, Bahia Brazil

**Keywords:** Stroke, Somatosensory stimulation, Transcranial magnetic stimulation

## Abstract

**Background:**

In healthy humans, somatosensory stimulation in the form of 2 h-repetitive peripheral afferent nerve stimulation (SS) increases excitability of the contralateral motor cortex. In this preliminary study, we explored effects of SS on excitability to transcranial magnetic stimulation (TMS) in patients with unilateral cerebellar infarcts and age-matched controls.

**Methods:**

Ten patients with infarcts in one cerebellar hemisphere and six age-matched controls participated in the study. Each subject participated in one session of active, and one session of sham SS delivered to the median nerve ipsilateral to the cerebellar infarct in patients, and to the homologous nerve in controls. Before and after each session, the following TMS measures were performed: resting motor threshold (rMT), motor evoked potentials (MEPs), short-interval intracortical inhibition (SICI) and short-interval intracortical facilitation (SICF). Amplitudes of motor evoked potentials were normalized to amplitudes of supramaximal M responses (MEP/M ratios).

**Results:**

In the control group, there was a significant increase in rMT, and a significant increase in MEP/M ratios after active, but not after sham SS. There were no significant differences in rMT or MEP/M ratios in the group of patients after active or sham SS. There were no significant differences in SICI or SICF after active or sham SS in either group.

**Conclusion:**

Consistent with results reported in rodents, these preliminary findings suggest for the first time in humans, that normal cerebellar activity is required so that SS can modulate excitability of the sensorimotor cortex.

## Background

In rodents, somatosensory stimulation (SS) enhances excitability of the contralateral motor cortex. This effect is decreased by down-regulation of cerebellar function, indicating that responsiveness to SS can be modulated by the cerebellum [1-4].

In healthy humans, SS in the form of 2 h-repetitive peripheral afferent nerve stimulation increases excitability of the contralateral motor cortex, reflected by increase in amplitudes of motor potentials evoked by transcranial magnetic stimulation (TMS) [5,6].

In both animals and humans, there is evidence that the effect of SS on cortical excitability is mediated at a supraspinal level [6-8], but the exact neural structures and pathways involved are still unclear. We explored effects of SS in the form of median nerve stimulation, in patients with infarcts affecting one cerebellar hemisphere, and in age-matched controls.

## Results

**Figure 1:**
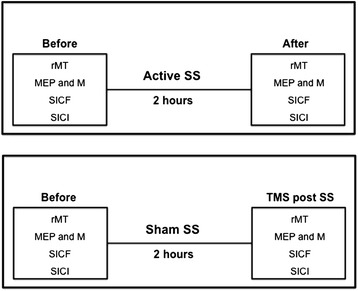
**Experimental design.** Each patient was submitted to two separate experimental sessions (active somatosensory stimulation, Active SS; sham somatosensory stimulation, Sham SS). In both sessions, resting motor thresholds (rMT), motor evoked potentials (MEPs), short-interval intracortical facilitation (SICF), short-interval intracortical inhibition (SICI) and supramaximal M response amplitudes were measured before and after somatosensory stimulation.

Before and after one session of active or sham SS (Figure 1), the following TMS measures were performed: resting motor threshold (rMT), motor evoked potentials (MEPs), short-interval intracortical inhibition (SICI) and short-interval intracortical facilitation (SICF). Amplitudes of motor evoked potentials were normalized to amplitudes of supramaximal M responses (MEP/M ratios).

Table 1 shows results of rMT, SICI and SICF.

**Table 1:** **Corticomotor excitability in patients and controls before and after active and sham sessions**

**Measure**	**Pre active**	**Post active**	**Pre sham**	**Post sham**
***Patients***				
**rMT (%s.o.)**	50.7 ± 10.8	51.2 ± 11.1	51.4 ± 11.8	51.3 ± 12.2
**SICI (%)**	85.1 ± 81.2	70.4 ± 41.4	64.2 ± 23.7	82 ± 53.8
**SICF (%)**	181.8 ± 107.4	166.3 ± 43.4	152.8 ± 56.4	177.9 ± 78.2
***Controls***				
**rMT (%s.o.)**	51.5 ± 8.5	52.7 ± 8.7*	50 ± 6.7	49.2 ± 7.7
**SICI (%)**	49 ± 27.7	58.8 ± 24.8	38.8 ± 24.5	46.9 ± 35
**SICF (%)**	204.2 ± 91.1	135.2 ± 62.3	202.8 ± 128.8	155 ± 52.5

rMT: resting motor threshold; s.o.: stimulator’s output; SICI: short-interval intracortical inhibition; SICF: short-interval intracortical facilitation; pre Active and post Active, pre Sham and post Sham: before and after repetitive peripheral stimulation in the active and sham sessions (means ± standard deviations). The asterisk indicates a significant difference (p ≤ 0.05).

All subjects reported intense paresthesias in the median nerve territory during the 2-h period of active SS, and none reported paresthesias during the session of sham SS. Table 2 shows initial stimulus intensities in each patient. Initial stimulation intensities were (average ± standard deviation), 48.5 ± 24.8 Volts (V) in patients and 64.2 ± 9.3 V in controls. There was no significant difference in initial intensities between the two groups (p = 0.368).

**Table 2:** **Characteristics of patients with cerebellar infarcts**

**Patient**	**Age (y)**	**Gender**	**Handedness**	**SARA**	**NIHSS**	**Arterial territory**	Stimulus intensity (V)*
1	63	F	R	1	0	PICA	70
2	49	M	R	6	2	PICA	80
3	39	F	L	11	4	SUCA	20
4	56	M	R	1	0	PICA	40
5	48	M	R	4.5	0	PICA	60
6	41	M	R	0	0	PICA	70
7	49	M	R	2	2	SUCA	50
8	21	F	R	1	0	PICA	4,5
9	55	M	R	0	0	PICA	20
10	53	M	R	9.5	3	PICA	70

y = years; F = female; M = male; R = right; L = left; SARA = scale for the assessment and rating of ataxia; NIHSS = National Institutes of Health Stroke Scale; PICA = posterior inferior cerebellar artery; SUCA = superior cerebellar artery; *initial stimulus intensity in the active somatosensory stimulation session (see text); V = Volts.

### Resting motor threshold

In patients, there were no significant effects of SESSION (active or sham SS), TIME (before or after stimulation) or interaction SESSION*TIME (p > 0.05) in regard to rMT.

In controls, there was a significant effect of SESSION (F = 7.2; p = 0.044), without significant effect of TIME or interaction SESSION*TIME. Post-hoc analysis showed a significant increase in rMT after active (p = 0.013), but not after sham SS (p = 0.526).

### MEP/M ratios (normalized MEP amplitudes)

**Figure 2:**
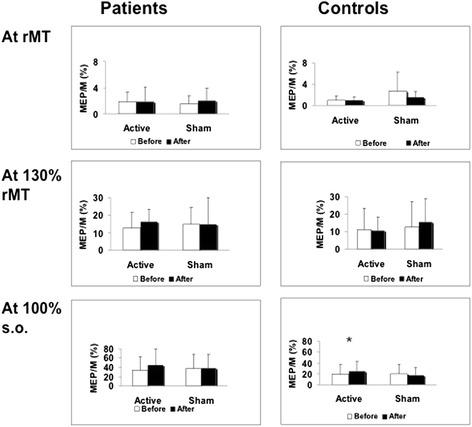
**Amplitudes of motor evoked potentials (MEP) expressed relative to the maximal peripheral M response peak-to-peak amplitudes (MEP/M, %), before and after the active and sham sessions, in the group of patients with cerebellar infarcts (left) and in the control group (right), at intensities corresponding to resting motor threshold (rMT), 130% rMT, and 100% of the stimulator’s output (s.o.).** The asterisk indicates a significant difference (p ≤ 0.05).

Figure 2 shows results in the active and control groups. Data from one subject were excluded due to technical factors.

In controls, there was a significant interaction of SESSION, TIME and INTENSITY (rMT, 130% rMT, and 100% of the stimulator’s output (s.o.); F = 6.7; p = 0.03). As expected, MEP/M ratios were greater at higher stimulation intensities. Post-hoc analysis showed a significant increase in MEP/M ratios at the intensity of 100% of the s.o. after active (p = 0.028), but not after sham SS (p = 0.452). There were no significant changes at the other two intensities of stimulation (p > 0.05).

In patients, there was only a significant effect of INTENSITY (F = 10.7, p = 0.009), with no significant effects of TIME, SESSION or any other significant interactions (p > 0.05).

### SICI and SICF

There were no significant effects of SESSION or TIME, nor interactions SESSION x TIME for SICI or SICF, in either group after active or sham SS (p > 0.05).

## Discussion

The main result of this preliminary study was the increase in MEP/M ratios after SS in controls but not in patients with unilateral cerebellar infarcts in the chronic phase, in line with results obtained in animals at an acute stage after downregulation of cerebellar outputs [1,2]. This result supports the hypothesis that normal cerebellar activity is required so that SS can modulate excitability of the sensorimotor cortex.

SS inputs reach the sensorimotor cortex by thalamocortical pathways, and reach the cerebellum by the spinocerebellar and spinoolivocerebellar tracts. The capacity of the cerebellum to process sensory information is underscored by estimates that, for each efferent axon that leaves the cerebellum, there are more than 40 afferent axons [9]. Cerebellar outputs project to the primary sensorimotor cortex mainly through the cerebellothalamocortical pathway.

It has been hypothesized that SS in the form of repetitive nerve stimulation, as performed in the present study, may enhance cortical excitability by augmenting effectiveness of the thalamocortical pathway [10]. It has also been suggested that, in order for this phenomenon to occur, functional integrity of cerebellothalamocortical projections is required. Integrity of axons originated in the interpositus nucleus is considered particularly important [1,3].

This concept has been strengthened by animal studies. In rodents, SS in the form of sciatic nerve stimulation at 10 Hz for one [1] or two [3] hours enhances motor potentials evoked by cortical electrical or magnetic stimulation of the motor cortex. Ipsilateral cerebellar intranuclear administration of alcohol, the sodium channel blocker tetrodotoxin, or electrical stimulation of cerebellar nuclei impair the enhancement of excitability by SS. In addition, modulation of primary sensorimotor cortex excitability by conditioning SS inputs is diminished in the acute fase after hemicerebellectomy in rats [4].

In this preliminary study, SS in the form of 2-h active repetitive median nerve electrical stimulation did not lead to significant changes in excitability to TMS in subjects with cerebellar infarcts, but was associated with significant increase in normalized MEP amplitudes from thenar muscles in controls, compared to sham stimulation.

Our patients were in the chronic phase (>4 months) after cerebellar infarcts. Because it has been described that corticomotor excitability to TMS is dynamic over time after a cerebellar infarct [11-13], it remains to be determined whether the observed effect may also be present at early stages after stroke, and whether it becomes more or less intense in the subsiding weeks and months, when responsiveness to cerebellar inputs may undergo plastic changes in the cortex or thalamus.

The increase in normalized MEP amplitudes was observed only at stimulus intensities corresponding to 100% of the s.o. Mechanisms underlying this phenomenon are not entirely clear. The increase in normalized MEP amplitudes of hand muscles at greater TMS intensities may be explained by recruitment of additional cortical neurons or spinal motor neurons, by evocation of multiple components of the corticospinal volley, or by enhancement of synchronization of spinal motor neurons [14].

In a previous study in healthy subjects, SS in the form of 2-h ulnar nerve stimulation led to increased normalized amplitudes of MEPs recorded at 120% rMT [5]. MEPs were recorded before and after active SS, and sham stimulation was not performed. In another study in which both active ulnar nerve stimulation and sham SS were applied, enhancement of normalized MEP amplitudes after active SS was more pronounced at greater TMS intensities, in line with our results [6]. Normalized MEPs were recorded before and after both interventions at TMS intensities ranging from below rMT up to 200% rMT [6]. Increase in normalized MEP amplitudes was noticed in 8/10 subjects at a TMS intensity of 140% rMT, but not at intensities of 130% rMT or lower. It is known that MEP amplitudes are less variable/more stable when recorded at greater TMS intensities [15]. We hypothesize that lower variability in MEP amplitudes recorded at higher TMS intensities is associated with greater power to detect changes in normalized MEP amplitudes after interventions such as SS, compared to MEP amplitudes registered at lower TMS intensities.

In addition, in contrast with studies that did not report changes in rMT after 2 h-ulnar nerve stimulation [5,6], in our control group there was a slight increase in rMT after the active session, but not after the sham session. It is possible that technical factors may explain these discrepancies: stimulated nerves (median *versus* ulnar), target muscles (*abductor digiti brevis,* ADM, *versus* thenar muscles) and sham intervention (no stimulation *versus* stimulation below sensory threshold). rMT seems to reflect membrane excitability and local density of a central core of excitatory interneurons and corticospinal neurons (targeted at the “hot spot”), and also of small spinal cord neurons [16]. MEP/M ratios reflect the extent of activation of the spinal motor neuron pool by a single TMS pulse at a given stimulus intensity [17]. Increases in MEP/M ratios at greater intensities of stimulation reflect progressive recruitment of less excitable or surrounding neurons in relation to the “hot spot” [16].

Even though under some circumstances, increase in rMT are accompanied by decrease in MEP/M ratios for a given target muscle (for instance, after lesions of the motor cortex or the corticospinal tract), interventions that modulate MEP amplitudes may not affect rMT and vice-versa [18]. It has been argued that modulation of MEP amplitudes by sensory input may reflect synaptic plasticity (i.e., lasting enhancement of synaptic transmission through long-term potentiation or long-term depression-like mechanisms) while changes in rMT would reflect intrinsic plasticity (i.e., excitability mediated by alterations in conductances of membrane ion channels) [18-20]. The increase in rMT and decrease in MEP/M at 100% rMT in the present study may reflect different effects of SS in the form of median nerve stimulation on core and surrounding neurons in the sensorimotor cortex, or different effects of SS on intrinsic and synaptic plasticity. This hypothesis deserves further investigation in a larger sample of subjects.

The lack of modulation of SICI or SICF by SS in the form of median nerve stimulation is consistent with results obtained after ulnar nerve stimulation in healthy subjects [6]. Conversely, in patients with hemiparesis caused by single subcortical strokes, 2-h ulnar and median nerve stimulation was associated with decrease in SICI [21].

The main limitation of this study is its sample size, leading to insufficient power to evaluate between-group differences. In addition, we cannot exclude the possibility that imbalance in gender explains the difference in responsiveness to active SS, compared to sham SS. This is a hypothesis-generating study. Considering the large number of patients screened, we suggest that multicenter studies are needed to confirm our findings.

## Conclusions

In summary, these preliminary results suggest for the first time in humans that cerebellar activity is crucial for modulation of the motor cortex by afferent SS in the form of repetitive peripheral nerve stimulation. These findings are consistent with those reported in rodents and highlight the importance of interactions between somatosensory and cerebellar inputs in modulation of cortical excitability.

## Methods

### Subjects

Ten patients with cerebellar infarcts (Patient_group_) and six healthy controls (Control_group_) participated in the study.

Inclusion criteria for patients were: age, 18–80 years; first-ever unilateral cerebellar infarct >4 months, confirmed by computed tomography or magnetic resonance imaging of the head and without brain lesions in the frontal lobe, parietal lobe, brainstem, thalamus, contralateral cerebellar hemisphere or any part of the corticospinal tract. We opted to include patients > 4 months post-stroke in order to avoid dynamic changes in cortical excitability that may occur during earlier phases after cerebellar infarcts [11]. Exclusion Criteria were: other neurological or severe chronic diseases; shoulder pain or severe joint deformity; use of medications that interfere with cortical excitability [22]; contraindications to transcranial magnetic stimulation [23]; inability to understand, provide informed consent, or follow instructions of the study.

**Figure 3:**
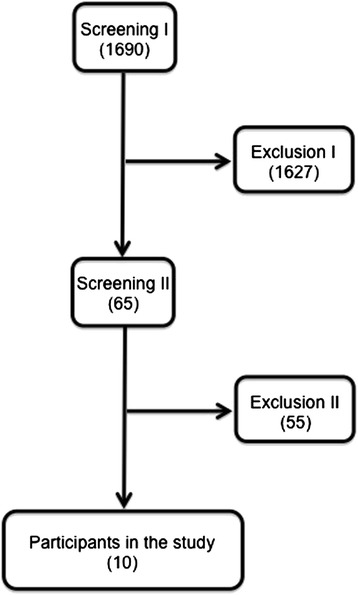
**Protocol’s flowchart.** The value in parentheses refers to the number of patients with stroke at each stage of the selection process. Screening I involved review of hospital charts and information from the Stroke Databank of our institution. Screening II involved telephone interviews. Patients who fulfilled criteria to participate in the study were then invited to come to the hospital for further evaluation of criteria and neurological evaluation. Only patients who fulfilled all inclusion criteria and did not present exclusion criteria participated in the study (n = 10).

Figure 3 shows the protocol’s flowchart.

Controls were included if they had no history of neurological disease, had a normal neurological examination, and their age, sex and handedness according to the Edinburgh Inventory [24] were comparable to those of the Patient_group_.

Median values of age (range) were 49 years (21–63) in patients and 53.5 years (39–64) in controls. Male/female ratios were 7/10 and 5/6 for patient and control subjects, respectively. Nine patients and six controls were right-handed according to the Edinburgh Inventory [24]. In patients, the median score in the SARA (scale for the assessment and rating of ataxia) was 1.5 (range, 0–11) and in the National Institute of Health stroke scale (NIHSS), 0 (range, 0–4).

SARA is an eight-item reliable and valid clinical scale used to assess ataxia; scores range from 0 (no ataxia) to 40 (most severe ataxia) [25]. NIHSS is a widely clinical scale that provides a quantitative measure of stroke-related neurologic deficit. Scores range from 0 to 42. Higher scores reflects more severe strokes [26].

The project was approved by our institutional Ethics Committee and all subjects provided written consent to participate.

### Experimental paradigm

Figure 1 shows the experimental protocol. This was a single-blind, randomized crossover, placebo-controlled proof-of-principle trial. Each subject participated to two sessions of active or sham SS. Each session lasted for about four hours (two hours of SS, and one hour of TMS measurements before and after SS). The order of the sessions was pseudorandomized across subjects. There was an interval of at least six days between the two sessions.

### Somatosensory stimulation (SS)

Each subject sat comfortably in an armchair with eyes open and arms at rest. Two silver surface electrodes connected to a stimulator were placed on the optimum point for stimulation of the median nerve on the wrist of the affected upper limb, with the cathode placed proximally as previously described [27,28].

Initially, the minimum intensity of stimulation at which patients reported paresthesias in the median nerve cutaneous territory (sensory threshold, ST) was measured three times. Trains of electrical stimuli of 1 ms duration (frequency of pulses within the train: 10 Hz) were administered at 1 Hz for a period of two hours with a portable stimulator (Alfamedic Ltda., São Paulo, Brazil). The output of the stimulator ranges from 0 to 400 V.

In the active SS session, stimulus intensity was increased until the maximum at which patients reported strong paresthesias in the median nerve territory in the absence of pain, while compound muscle action potential amplitudes were below 100 μV in the *abductor pollicis brevis* muscle (APB). Background electromyography (EMG) activity recorded from surface electrodes in the APB was continuously monitored. During the 2-h period, sensations described by patients were checked every five minutes, because they can change at the same stimulus intensity, for example due to changes in skin impedance. If paresthesias decreased or if EMG activity above100 μV was noticed in the APB, stimulus intensities were adjusted accordingly throughout the 2-h period.

In the sham SS session, intensity was set to 10 V below sensory threshold [27,29].

### Transcranial magnetic stimulation

TMS was delivered through a figure-of-eight shaped magnetic coil (outside diameter 70 mm, maximum rate of change 22.5 × 103 T/s) connected to two 200^2^ Magstim stimulators through a Bistim^2^ module (The Magstim Company, Dyfed, UK). The magnetic coil was placed tangentially to the scalp, with the intersection of both wings at a 45° angle with the midline to optimally stimulate the motor cortex [30]. Electromyographic (EMG) activity was recorded from surface electrodes placed over the APB. EMG responses were amplified (1000), filtered (2 Hz-2 kHz) and recorded on a computerized data acquisition system built with the LabVIEW graphical programming language (sampling rate 5 kHz) [31]. Its conditional triggering’ feature was used to deliver TMS stimuli only when the APB was relaxed. Relaxation was defined as EMG activity at baseline < 50 microvolts (μV) peak-to-peak amplitude for at least 1 s.

TMS measurements were obtained after identification of the optimal position (hot spot) of the APB muscle. To define the APB hot spot, we initially set the intensity to 50% of the maximum stimulator’s output, and then decreased it in 5% steps, moving the coil anteroposteriorly and mediolaterally in steps of 0.5 cm. After identification of points at which no MEPs higher than 100 μV were elicited in all directions, the intensity was decreased in 1-2% steps. The search was repeated iteratively until 3 MEPs were observed out of 3 trials at a given position, while stimulation of adjacent positions did not evoke reliable MEPs on 3 trials. If no MEPs were evoked at any position at a given intensity, while at an intensity 1% higher, 3 MEPs were still observed out of 3 trials in more than one point, the ‘hot spot’ was defined as the position in which the largest mean MEP amplitude was detected.

In patients, the cerebral hemisphere contralateral to the cerebellar infarct was stimulated. The homologous hemisphere was stimulated in controls.

The following TMS measurements were performed before and after active or sham SS:

- Resting motor threshold (rMT), defined as the minimum TMS intensity required to elicit at least three out of six motor evoked potentials (MEP) ≥50 μV in consecutive trials at rest. TMS stimulus intensities were expressed relative to rMT measured from the APB [32].
- MEP amplitudes recorded at intensities corresponding to rMT, 130% rMT, and 100% (maximal intensity) of the stimulator’s output. Ten trials were recorded at each stimulation intensity. M responses were obtained by supramaximal stimulation of the median nerve at the wrist (10 trials). MEP amplitudes were expressed relative to the maximal peripheral M response peak-to-peak amplitudes (MEP/M, %). This measurement controls for differences in muscle bulk and electrode position across subjects and reflects the extent of activation of the spinal motor neuron pool of a target muscle by a single TMS pulse at a given stimulus intensity [15,17].
- Short-interval intracortical facilitation (SICF), short-interval intracortical inhibition (SICI), determined with a paired-pulse protocol [33]. The intensity of the test stimulus (TS) was that required to evoke MEPs (MEP_TS_) of approximately 0.5–1 mV. The intensity of the conditioning stimulus was 80% of the APB rMT. The order of presentation of inhibitory (2 ms) and facilitatory (10 ms) trials as well as test stimuli alone was randomized. Sixteen trials were recorded for each interstimulus interval (2 ms and 10 ms), and sixteen trials were recorded after administration of TS alone.

### Statistical analysis

Repeated-measures analysis of variance was performed separately for the two groups, with factors SESSION (active or sham) and TIME (before and after stimulation). For MEP/M ratios, INTENSITY OF STIMULATION was an additional factor. Post-hoc t-tests were not corrected for multiple comparisons, given the exploratory nature of this study. Mann–Whitney tests were used to compare initial stimulation intensities between the two groups.

## Abbreviations

APB: *Abductor pollicis brevis* muscle
EMG: Electromyography
MEP: Motor evoked potentials
MEP/M ratios: Amplitudes of motor evoked potentials normalized to amplitudes of supramaximal M responses
μV: Microvolts
NIHSS: National Institutes of Health stroke scale
rMT: Resting motor threshold
SARA: Scale for the assessment and rating of ataxia
SICI: Short-interval intracortical inhibition
SICF: Short-interval intracortical facilitation
SS: Somatosensory stimulation
s.o.: Stimulator’s output
TMS: Transcranial magnetic stimulation
TS: Test stimulus
V: Volts
