# Erratum to: Effects of somatosensory stimulation on corticomotor excitability in patients with unilateral cerebellar infarcts and healthy subjects - preliminary results

**DOI:** 10.1186/s40673-016-0048-0

**Published:** 2016-04-25

**Authors:** Suzete Nascimento Farias da Guarda, Adriana Bastos Conforto

**Affiliations:** Hospital das Clínicas/São Paulo University, São Paulo, Brazil; Hospital São Rafael, Salvador, Brazil; Instituto Israelita de Ensino e Pesquisa Albert Einstein, São Paulo, Brazil; Rua Waldemar Falcão n 1547 ap 1201 Horto Florestal 40.295-010, Salvador, Bahia Brazil

## Erratum

Following publication of this work [1] the authors noticed a mistake within the article’s discussion section. The original mistake and resulting clarification are outlined below:

In the discussion section (paragraph 10) where it is written:

“The increase in rMT and decrease in MEP/M at 100 % rMT in the present study…”

It should read:

“The increase in rMT and increase in MEP/M at 100 % rMT in the present study…”
